# Supplementary material for: Association between COVID-19 vaccination and sudden death in apparently healthy younger individuals: A population-based case-control study
Source: PLoS Med. 2026 Mar 19;23(3):e1004924. doi: 10.1371/journal.pmed.1004924 (PMC13001984; doi:10.1371/journal.pmed.1004924)
Supplement: S1 Table — (DOCX) [file pmed.1004924.s002.docx]

**S1 Table. ICD-10 diagnostic codes used for defining a case.**

| **Codes for inclusion** | |
| --- | --- |
| I46 | Cardiac arrest |
| I47.0 | Re-entry ventricular arrhythmia |
| I47.2 | Ventricular tachycardia |
| I49.0 | Ventricular fibrillation and flutter |
| I49.8 | Other specified cardiac arrhythmias |
| I49.9 | Cardiac arrhythmia, unspecified |
| R96 | Other sudden death, cause unknown |
| R98 | Unattended death |
| **Codes for exclusion** | |
| F00-F99 | Mental and behavioural disorders |
| S00-T98 | Injury, poisoning and certain other consequences of external causes |
| V01-V99 | Transport accidents |
| W00-X59 | Other external causes of accidental injury |
| X60-X84 | Intentional self-harm |
| X85-Y09 | Assault |
| Y10-Y34 | Event of undetermined intent |
| Y35-Y36 | Legal intervention and operations of war |
| Y40-Y84 | Complications of medical and surgical care |
| Y85-Y89 | Sequelae of external causes of morbidity and mortality |
| Y90-Y98 | Supplementary factors related to causes of morbidity and mortality classified elsewhere |
