# Supplementary material for: Association between COVID-19 vaccination and sudden death in apparently healthy younger individuals: A population-based case-control study
Source: PLoS Med. 2026 Mar 19;23(3):e1004924. doi: 10.1371/journal.pmed.1004924 (PMC13001984; doi:10.1371/journal.pmed.1004924)
Supplement: S2 Table — The data are presented after dividing the cohort into two groups—people who met the definition for being a case, and those who were eligible for being selected as controls. Please note that the cases are not matched to controls in this table. (DOCX) [file pmed.1004924.s003.docx]

**S2 Table.** **Baseline characteristics of the residents of Ontario, Canada who met criteria for inclusion in the study. The data are presented after dividing the cohort into two groups - people who met the definition for being a case, and those who were eligible for being selected as controls. Please note that the cases are not matched to controls in this table.**

| **Variable** | **Cases** | **Controls** | **Std. Diff*** |
| --- | --- | --- | --- |
|  | N=4,963 | N=6,360,488 |  |
| **Age, mean ± SD, years** | 35.1 ± 10.5 | 31.0 ± 10.8 | 0.39 |
| **Age, median (Q1-Q3), years** | 36 (27 - 45) | 31 (22 - 40) | 0.38 |
| **Aged 12-18 years, n(%)** | 374 (7.5%) | 1,046,100 (16.4%) | 0.28 |
| **Aged 19-30 years, n(%)** | 1,347 (27.1%) | 2,036,926 (32.0%) | 0.11 |
| **Aged 31-40 years, n(%)** | 1,381 (27.8%) | 1,778,628 (28.0%) | <0.01 |
| **Aged 41-50 years, n(%)** | 1,861 (37.5%) | 1,498,834 (23.6%) | 0.31 |
| **Male sex, n(%)** | 3,690 (74.4%) | 3,172,848 (49.9%) | 0.52 |
| **Public health unit region** | | | |
| **Central East, n(%)** | 397 (8.0%) | 364,121 (5.7%) | 0.09 |
| **Central West, n(%)** | 957 (19.3%) | 1,237,760 (19.5%) | <0.01 |
| **Durham, n(%)** | 244 (4.9%) | 320,427 (5.0%) | 0.01 |
| **Eastern, n(%)** | 348 (7.0%) | 324,433 (5.1%) | 0.08 |
| **Northern, n(%)** | 495 (10.0%) | 301,116 (4.7%) | 0.20 |
| **Ottawa, n(%)** | 312 (6.3%) | 466,312 (7.3%) | 0.04 |
| **Peel, n(%)** | 411 (8.3%) | 731,949 (11.5%) | 0.11 |
| **Southwest, n(%)** | 688 (13.9%) | 701,091 (11.0%) | 0.09 |
| **Toronto, n(%)** | 824 (16.6%) | 1,336,789 (21.0%) | 0.11 |
| **York, n(%)** | 261 (5.3%) | 551,248 (8.7%) | 0.13 |
| **Missing data, n(%)** | 26 (0.5%) | 25,242 (0.4%) | 0.02 |
| **Neighborhood income quintile** | | | |
| **1 (Lowest), n(%)** | 1,330 (26.8%) | 1,201,210 (18.9%) | 0.19 |
| **2, n(%)** | 1,015 (20.5%) | 1,224,009 (19.2%) | 0.03 |
| **3, n(%)** | 974 (19.6%) | 1,296,797 (20.4%) | 0.02 |
| **4, n(%)** | 864 (17.4%) | 1,326,797 (20.9%) | 0.09 |
| **5 (Highest), n(%)** | 750 (15.1%) | 1,284,295 (20.2%) | 0.13 |
| **Missing data, n(%)** | 30 (0.6%) | 27,380 (0.4%) | 0.02 |
| **Neighborhood average number of persons per dwelling quintile** | | | |
| **1 (Lowest), n(%)** | 1,043 (21.0%) | 1,014,679 (16.0%) | 0.13 |
| **2, n(%)** | 999 (20.1%) | 946,916 (14.9%) | 0.14 |
| **3, n(%)** | 632 (12.7%) | 784,607 (12.3%) | 0.01 |
| **4, n(%)** | 1,045 (21.1%) | 1,479,335 (23.3%) | 0.05 |
| **5 (Highest), n(%)** | 874 (17.6%) | 1,565,709 (24.6%) | 0.17 |
| **Missing data, n(%)** | 370 (7.5%) | 569,242 (8.9%) | 0.05 |
| **Neighborhood quintile by proportion of people who self-identify as visible minority quintile** | | | |
| **1 (Lowest), n(%)** | 980 (19.7%) | 800,118 (12.6%) | 0.20 |
| **2, n(%)** | 945 (19.0%) | 931,686 (14.6%) | 0.12 |
| **3, n(%)** | 811 (16.3%) | 1,050,569 (16.5%) | <0.01 |
| **4, n(%)** | 936 (18.9%) | 1,355,724 (21.3%) | 0.06 |
| **5 (Highest), n(%)** | 920 (18.5%) | 1,652,870 (26.0%) | 0.18 |
| **Missing data, n(%)** | 371 (7.5%) | 569,521 (9.0%) | 0.05 |
| **Neighborhood quintile by proportion employed in sales/trades/manufacturing/agriculture** | | | |
| **1 (Lowest), n(%)** | 666 (13.4%) | 1,244,310 (19.6%) | 0.17 |
| **2, n(%)** | 875 (17.6%) | 1,372,432 (21.6%) | 0.10 |
| **3, n(%)** | 939 (18.9%) | 1,162,161 (18.3%) | 0.02 |
| **4, n(%)** | 1,037 (20.9%) | 1,065,535 (16.8%) | 0.11 |
| **5 (Highest), n(%)** | 1,075 (21.7%) | 946,479 (14.9%) | 0.18 |
| **Missing data, n(%)** | 371 (7.5%) | 569,571 (9.0%) | 0.05 |
| **Asthma, n(%)** | 1,003 (20.2%) | 1,077,563 (16.9%) | 0.08 |
| **Hypertension, n(%)** | 424 (8.5%) | 254,507 (4.0%) | 0.19 |
| **History of mood or anxiety disorder in the past 5 years, n(%)** | 272 (5.5%) | 115,232 (1.8%) | 0.20 |
| **Influenza vaccination in past year, n(%)** | 607 (12.2%) | 1,254,549 (19.7%) | 0.21 |
| **Number of COVID-19 vaccine doses received as of index date** | | | |
| **0, n(%)** | 4,712 (94.9%) | 5,930,599 (93.2%) | 0.07 |
| **1, n(%)** | 180 (3.6%) | 322,837 (5.1%) | 0.07 |
| **≥2, n(%)** | 71 (1.4%) | 107,052 (1.7%) | 0.02 |

* std = standardized difference
