# Supplementary material for: Association between COVID-19 vaccination and sudden death in apparently healthy younger individuals: A population-based case-control study
Source: PLoS Med. 2026 Mar 19;23(3):e1004924. doi: 10.1371/journal.pmed.1004924 (PMC13001984; doi:10.1371/journal.pmed.1004924)
Supplement: S3 Table — (DOCX) [file pmed.1004924.s004.docx]

**S3 Table. Baseline characteristics of matched cases and controls aged <40 years.**

| **Variable** | **Cases** | **Controls** | **Std. Diff*** |
| --- | --- | --- | --- |
|  | N=2,849 | N=14,245 |  |
| **Age, mean ± SD, years** | 28.10 ± 7.15 | 28.10 ± 7.15 | 0 |
| **Age, median (IQR), years** | 29 (22-34) | 29 (22-34) | 0 |
| **Aged 12-18 years, n(%)** | 346 (12.1%) | 1,730 (12.1%) | 0 |
| **Aged 19-30 years, n(%)** | 1,309 (45.9%) | 6,545 (45.9%) | 0 |
| **Aged 31-40 years, n(%)** | 1,194 (41.9%) | 5,970 (41.9%) | 0 |
| **Male sex, n(%)** | 2,124 (74.6%) | 10,620 (74.6%) | 0 |
| **Public health unit region** | | | |
| **Central East, n(%)** | 222 (7.8%) | 1,090 (7.7%) | 0.01 |
| **Central West, n(%)** | 533 (18.7%) | 2,662 (18.7%) | 0 |
| **Durham, n(%)** | 141 (4.9%) | 693 (4.9%) | 0 |
| **Eastern, n(%)** | 193 (6.8%) | 1,012 (7.1%) | 0.01 |
| **Northern, n(%)** | 265 (9.3%) | 1,324 (9.3%) | 0 |
| **Ottawa, n(%)** | 179 (6.3%) | 871 (6.1%) | 0.01 |
| **Peel, n(%)** | 258 (9.1%) | 1,290 (9.1%) | 0 |
| **Southwest, n(%)** | 393 (13.8%) | 1,966 (13.8%) | 0 |
| **Toronto, n(%)** | 513 (18.0%) | 2,565 (18.0%) | 0 |
| **York, n(%)** | 143 (5.0%) | 727 (5.1%) | 0 |
| **Missing data, n(%)** | 9 (0.3%) | 45 (0.3%) | 0 |
| **Neighbourhood income quintile** | | | |
| **1 (Lowest), n(%)** | 795 (27.9%) | 3,975 (27.9%) | 0 |
| **2, n(%)** | 582 (20.4%) | 2,910 (20.4%) | 0 |
| **3, n(%)** | 543 (19.1%) | 2,715 (19.1%) | 0 |
| **4, n(%)** | 499 (17.5%) | 2,495 (17.5%) | 0 |
| **5 (Highest), n(%)** | 418 (14.7%) | 2,090 (14.7%) | 0 |
| **Missing data, n(%)** | 12 (0.4%) | 60 (0.4%) | 0 |
| **Neighborhood average number of persons per dwelling quintile** | | | |
| **1 (Lowest), n(%)** | 582 (20.4%) | 2,747 (19.3%) | 0.03 |
| **2, n(%)** | 564 (19.8%) | 2,668 (18.7%) | 0.03 |
| **3, n(%)** | 345 (12.1%) | 1,842 (12.9%) | 0.02 |
| **4, n(%)** | 605 (21.2%) | 3,168 (22.2%) | 0.02 |
| **5, n(%)** | 526 (18.5%) | 2,688 (18.9%) | 0.01 |
| **Missing, n(%)** | 227 (8.0%) | 1,132 (7.9%) | 0 |
| **Neighborhood quintile by proportion of people who self-identify as visible minority quintile** | | | |
| **1 (Lowest), n(%)** | 541 (19.0%) | 2,743 (19.3%) | 0.01 |
| **2, n(%)** | 519 (18.2%) | 2,437 (17.1%) | 0.03 |
| **3, n(%)** | 457 (16.0%) | 2,338 (16.4%) | 0.01 |
| **4, n(%)** | 520 (18.3%) | 2,647 (18.6%) | 0.01 |
| **5 (Highest), n(%)** | 584 (20.5%) | 2,948 (20.7%) | 0 |
| **Missing, n(%)** | 228 (8.0%) | 1,132 (7.9%) | 0 |
| **Neighborhood quintile by proportion employed in sales/trades/manufacturing/agriculture** | | | |
| **1 (Lowest)**, **n(%)** | 398 (14.0%) | 1,939 (13.6%) | 0.01 |
| **2, n(%)** | 498 (17.5%) | 2,624 (18.4%) | 0.02 |
| **3, n(%)** | 512 (18.0%) | 2,746 (19.3%) | 0.03 |
| **4, n(%)** | 589 (20.7%) | 2,851 (20.0%) | 0.02 |
| **5 (Highest), n(%)** | 624 (21.9%) | 2,953 (20.7%) | 0.03 |
| **Missing, n(%)** | 228 (8.0%) | 1,132 (7.9%) | 0 |
| **Asthma, n(%)** | 692 (24.3%) | 2,939 (20.6%) | 0.09 |
| **Hypertension, n(%)** | 88 (3.1%) | 223 (1.6%) | 0.10 |
| **History of mood or anxiety disorder in the past 5 years, n(%)** | 185 (6.5%) | 244 (1.7%) | 0.24 |
| **Influenza vaccination in past year, n(%)** | 287 (10.1%) | 2,154 (15.1%) | 0.15 |
| **Number of COVID-19 vaccine doses received as of index date** | | | |
| **0, n(%)** | 1,017 (35.7%) | 3,467 (24.3%) | 0.25 |
| **1, n(%)** | 194 (6.8%) | 869 (6.1%) | 0.03 |
| **≥2, n(%)** | 1,638 (57.5%) | 9,909 (69.6%) | 0.25 |
| **Received any COVID-19 vaccine before index date, n(%)** | 1,832 (64.3%) | 10,778 (75.7%) | 0.25 |
| **Received COVID-19 vaccine within 6 weeks before index date, n(%)** | 176 (6.2%) | 1,294 (9.1%) | 0.11 |
| **Received ≥1 dose of any mRNA vaccine, n(%)** | 1,829 (64.2%) | 10,752 (75.5%) | 0.25 |
| **Received ≥1 dose of Pfizer/BioNTech Comirnaty vaccine, n(%)** | 1,454 (44.4%) | 8,964 (62.9%) | 0.24 |
| **Received ≥1 dose of Moderna Spikevax vaccine, n(%)** | 744 (26.1%) | 6,638 (29.1%) | 0.07 |
| **Received ≥1 dose of AstraZeneca Vaxzevria vaccine, n(%)** | 9 (0.3%) | 25 (0.2%) | 0.03 |
| **Recent SARS-CoV-2 PCR test before case death date** | | | |
| **Never tested positive before, n(%)** | 2,598 (91.2%) | 13,050 (91.6%) | 0.02 |
| **Remote prior positive test (>90 days), n(%)** | 191 (6.7%) | 1,066 (7.5%) | 0.03 |
| **Recent prior positive test (≤90 days), n(%)** | 60 (2.1%) | 129 (0.9%) | 0.10 |
| **Number of SARS-CoV-2 PCR tests prior to case death date** | | | |
| **Mean ± SD** | 1.40 ± 3.24 | 1.15 ± 2.82 | 0.08 |
| **Median (IQR)** | 1 (0-2) | 0 (0-1) | 0.11 |

* std = standardized difference
