# Supplementary material for: Association between COVID-19 vaccination and sudden death in apparently healthy younger individuals: A population-based case-control study
Source: PLoS Med. 2026 Mar 19;23(3):e1004924. doi: 10.1371/journal.pmed.1004924 (PMC13001984; doi:10.1371/journal.pmed.1004924)
Supplement: S4 Table — (S4_Table.DOCX) [file pmed.1004924.s005.docx]

**S4 Table. Baseline characteristics of matched cases and controls excluding opioid-related deaths.**

| **Variable** | **Cases** | **Controls** | **Std. Diff*** |
| --- | --- | --- | --- |
|  | N=2,016 | N=10,080 |  |
| **Age, mean ± SD, years** | 35.48 ± 10.50 | 34.83 ± 10.49 | 0.06 |
| **Age, median (IQR), years** | 37 (27-45) | 36 (26-44) | 0.07 |
| **Aged 12-18 years, n(%)** | 138 (6.8%) | 690 (6.8%) | 0 |
| **Aged 19-30 years, n(%)** | 537 (26.6%) | 2,685 (26.6%) | 0 |
| **Aged 31-40 years, n(%)** | 555 (27.5%) | 6,775 (27.5%) | 0 |
| **Aged 41-50 years, n(%)** | 786 (39.0%) | 3,930 (39.0%) | 0 |
| **Male sex, n(%)** | 1,476 (73.2%) | 7,380 (73.2%) | 0 |
| **Public health unit region** | | | |
| **Central East, n(%)** | 139 (6.9%) | 654 (6.5%) | 0.02 |
| **Central West, n(%)** | 362 (18.0%) | 1,823 (18.1%) | 0 |
| **Durham, n(%)** | 107 (5.3%) | 530 (5.3%) | 0 |
| **Eastern, n(%)** | 145 (7.2%) | 767 (7.6%) | 0.02 |
| **Northern, n(%)** | 193 (9.6%) | 964 (9.6%) | 0 |
| **Ottawa, n(%)** | 130 (6.4%) | 645 (6.4%) | 0 |
| **Peel, n(%)** | 179 (8.9%) | 895 (8.9%) | 0 |
| **Southwest, n(%)** | 269 (13.3%) | 1,329 (13.2%) | 0 |
| **Toronto, n(%)** | 371 (18.4%) | 1,855 (18.4%) | 0 |
| **York, n(%)** | 116 (5.8%) | 593 (5.9%) | 0.01 |
| **Missing data, n(%)** | ≤5^1^ | 25 (0.2%) | 0 |
| **Neighborhood income quintile** | | | |
| **1 (Lowest), n(%)** | 535 (26.5%) | 2,675 (26.5%) | 0 |
| **2, n(%)** | 422 (20.9%) | 2,110 (20.9%) | 0 |
| **3, n(%)** | 392 (19.4%) | 1,960 (19.4%) | 0 |
| **4, n(%)** | 340 (16.9%) | 1,700 (16.9%) | 0 |
| **5 (Highest), n(%)** | 320 (15.9%) | 1,600 (15.9%) | 0 |
| **Missing data, n(%)** | 7 (0.3%) | 35 (0.3%) | 0 |
| **Neighborhood average number of persons per dwelling quintile** | | | |
| **1 (Lowest), n(%)** | 394 (19.5%) | 1,940 (19.2%) | 0.01 |
| **2, n(%)** | 396 (19.6%) | 1,903 (18.9%) | 0.02 |
| **3, n(%)** | 241 (12.0%) | 1,328 (13.2%) | 0.04 |
| **4, n(%)** | 405 (20.1%) | 2,088 (20.7%) | 0.02 |
| **5, n(%)** | 408 (20.2%) | 2,040 (20.2%) | 0 |
| **Missing, n(%)** | 172 (8.5%) | 781 (7.7%) | 0.03 |
| **Neighborhood quintile by proportion of people who self-identify as visible minority quintile** | | | |
| **1 (Lowest), n(%)** | 391 (19.4%) | 1,952 (19.4%) | 0 |
| **2, n(%)** | 351 (17.4%) | 1,721 (17.1%) | 0.01 |
| **3, n(%)** | 314 (15.6%) | 1,651 (16.4%) | 0.02 |
| **4, n(%)** | 381 (18.9%) | 1,842 (18.3%) | 0.02 |
| **5 (Highest), n(%)** | 407 (20.2%) | 2,133 (21.1%) | 0.02 |
| **Missing, n(%)** | 172 (8.5%) | 781 (7.7%) | 0.03 |
| **Neighborhood quintile by proportion employed in sales/trades/manufacturing/agriculture** | | | |
| **1 (Lowest)**, **n(%)** | 280 (13.9%) | 1,473 (14.6%) | 0.02 |
| **2, n(%)** | 361 (17.9%) | 1,830 (18.2%) | 0.01 |
| **3, n(%)** | 357 (17.7%) | 1,873 (18.6%) | 0.02 |
| **4, n(%)** | 409 (20.3%) | 1,992 (19.8%) | 0.01 |
| **5 (Highest), n(%)** | 437 (21.7%) | 2,131 (21.1%) | 0.01 |
| **Missing, n(%)** | 172 (8.5%) | 781 (7.7%) | 0.03 |
| **Asthma, n(%)** | 366 (18.2%) | 1,681 (16.7%) | 0.04 |
| **Hypertension, n(%)** | 195 (9.7%) | 614 (6.1%) | 0.13 |
| **History of mood or anxiety disorder in the past 5 years, n(%)** | 114 (5.7%) | 168 (1.7%) | 0.21 |
| **Influenza vaccination in past year, n(%)** | 266 (13.2%) | 1,696 (16.8%) | 0.10 |
| **Number of COVID-19 vaccine doses received as of index date** | | | |
| **0, n(%)** | 784 (38.9%) | 2,921 (29.0%) | 0.21 |
| **1, n(%)** | 199 (9.9%) | 1,035 (10.3%) | 0.01 |
| **≥2, n(%)** | 1,033 (51.2%) | 6,124 (60.8%) | 0.19 |
| **Received any COVID-19 vaccine before index date, n(%)** | 1,232 (61.1%) | 7,159 (71.0%) | 0.21 |
| **Received COVID-19 vaccine within 6 weeks before index date, n(%)** | 258 (12.8%) | 1,787 (17.7%) | 0.14 |
| **Received ≥1 dose of any mRNA vaccine, n(%)** | 1,217 (60.4%) | 7,058 (70.0%) | 0.20 |
| **Received ≥1 dose of Pfizer/BioNTech Comirnaty vaccine, n(%)** | 896 (44.4%) | 5,500 (54.6%) | 0.20 |
| **Received ≥1 dose of Moderna Spikevax vaccine, n(%)** | 518 (25.7%) | 2,842 (28.2%) | 0.06 |
| **Received ≥1 dose of AstraZeneca Vaxzevria vaccine, n(%)** | 62 (3.1%) | 464 (4.6%) | 0.08 |
| **Recent SARS-CoV-2 PCR test before case death date** | | | |
| **Never tested positive before, n(%)** | 1,966 (97.5%) | 9,937 (97.6%) | 0 |
| **Remote prior positive test (>90 days), n(%)** | 20 (1.0%) | 132 (1.3%) | 0.03 |
| **Recent prior positive test (≤90 days), n(%)** | 30 (1.5%) | 111 (1.1%) | 0.03 |
| **Number of SARS-CoV-2 PCR tests prior to case death date** | | | |
| **Mean ± SD** | 0.61 ± 2.21 | 0.62 ± 2.16 | 0.01 |
| **Median (IQR)** | 0 (0-2) | 0 (0-1) | 0 |

* std = standardized difference

^1^ Cells with <6 individuals are suppressed to reduce the risk of re-identification as per ICES contractual obligations with data providers.
