# Supplementary material for: Association between COVID-19 vaccination and sudden death in apparently healthy younger individuals: A population-based case-control study
Source: PLoS Med. 2026 Mar 19;23(3):e1004924. doi: 10.1371/journal.pmed.1004924 (PMC13001984; doi:10.1371/journal.pmed.1004924)
Supplement: S5 Table — (DOCX) [file pmed.1004924.s006.docx]

**S5 Table. Baseline characteristics of matched cases and controls only including deaths within 24-hours of presenting to the hospital.**

| **Variable** | **Cases** | **Controls** | **Std. Diff*** |
| --- | --- | --- | --- |
|  | N=496 | N=2,480 |  |
| **Age, mean ± SD, years** | 37.42 ± 10.15 | 37.42 ± 10.14 | 0 |
| **Age, median (Q1-Q3), years** | 40 (30-46) | 40 (30-46) | 0 |
| **Aged 12-18 years, n(%)** | 34 (6.9%) | 170 (6.9%) | 0 |
| **Aged 19-30 years, n(%)** | 93 (18.8%) | 465 (18.8%) | 0 |
| **Aged 31-40 years, n(%)** | 129 (26.0%) | 645 (26.0%) | 0 |
| **Aged 41-50 years, n(%)** | 240 (48.4%) | 1,200 (48.4%) | 0 |
| **Male sex, n(%)** | 341 (68.8%) | 1,705 (68.8%) | 0 |
| **Public health unit region** | | | |
| **Central East, n(%)** | 27 (5.4%) | 125 (5.0%) | 0.02 |
| **Central West, n(%)** | 103 (20.8%) | 511 (20.6%) | 0 |
| **Durham, n(%)** | 27 (5.4%) | 136 (5.5%) | 0 |
| **Eastern, n(%)** | 36 (7.3%) | 197 (7.9%) | 0.03 |
| **Northern, n(%)** | 39 (7.9%) | 195 (7.9%) | 0 |
| **Ottawa, n(%)** | 31 (6.3%) | 149 (6.0%) | 0.01 |
| **Peel, n(%)** | 49 (9.9%) | 245 (9.9%) | 0 |
| **Southwest, n(%)** | 59 (11.9%) | 300 (12.1%) | 0.01 |
| **Toronto, n(%)** | 84 (16.9%) | 420 (16.9%) | 0 |
| **York, n(%)** | 39 (7.9%) | 192 (7.7%) | 0 |
| **Missing data, n(%)** | ≤5^1^ | 10 (0.4%) | 0 |
| **Neighbourhood income quintile** | | | |
| **1 (Lowest), n(%)** | 111 (22.4%) | 555 (22.4%) | 0 |
| **2, n(%)** | 97 (19.6%) | 485 (19.6%) | 0 |
| **3, n(%)** | 110 (22.2%) | 550 (22.2%) | 0 |
| **4, n(%)** | 94 (19.0%) | 470 (19.0%) | 0 |
| **5 (Highest), n(%)** | 82 (16.5%) | 410 (16.5%) | 0 |
| **Missing data, n(%)** | ≤5^1^ | 10 (0.4%) | 0 |
| **Neighborhood average number of persons per dwelling quintile** | | | |
| **1 (Lowest), n(%)** | 85 (17.1%) | 427 (17.2%) | 0 |
| **2, n(%)** | 101 (20.4%) | 463 (18.7%) | 0.04 |
| **3, n(%)** | 55 (11.1%) | 295 (11.9%) | 0.03 |
| **4, n(%)** | 106 (21.4%) | 548 (22.1%) | 0.02 |
| **5 (Highest), n(%)** | 108 (21.8%) | 535 (21.6%) | 0 |
| **Missing data, n(%)** | 41 (8.3%) | 212 (8.5%) | 0.01 |
| **Neighborhood quintile by proportion of people who self-identify as visible minority quintile** | | | |
| **1 (Lowest), n(%)** | 78 (15.7%) | 391 (15.8%) | 0 |
| **2, n(%)** | 86 (17.3%) | 396 (16.0%) | 0.04 |
| **3, n(%)** | 95 (19.2%) | 402 (16.2%) | 0.08 |
| **4, n(%)** | 86 (17.3%) | 510 (20.6%) | 0.08 |
| **5 (Highest), n(%)** | 110 (22.2%) | 569 (22.9%) | 0.02 |
| **Missing data, n(%)** | 41 (8.3%) | 212 (8.5%) | 0.01 |
| **Neighborhood quintile by proportion employed in sales/trades/manufacturing/agriculture** | | | |
| **1 (Lowest), n(%)** | 75 (15.1%) | 338 (13.6%) | 0.04 |
| **2, n(%)** | 85 (17.1%) | 520 (21.0%) | 0.10 |
| **3, n(%)** | 109 (22.0%) | 512 (20.6%) | 0.03 |
| **4, n(%)** | 97 (19.6%) | 481 (19.4%) | 0 |
| **5 (Highest), n(%)** | 89 (17.9%) | 417 (16.8%) | 0.03 |
| **Missing data, n(%)** | 41 (8.3%) | 212 (8.5%) | 0.01 |
| **Asthma, n(%)** | 100 (20.2%) | 394 (15.9%) | 0.11 |
| **Hypertension, n(%)** | 66 (13.3%) | 176 (7.1%) | 0.21 |
| **History of mood or anxiety disorder in the past 5 years, n(%)** | 21 (4.2%) | 47 (1.9%) | 0.14 |
| **Influenza vaccination in past year, n(%)** | 72 (14.5%) | 451 (18.2%) | 0.10 |
| **Number of COVID-19 vaccine doses received as of index date** | | | |
| **0, n(%)** | 131 (26.4%) | 528 (21.3%) | 0.12 |
| **1, n(%)** | 25 (5.0%) | 157 (6.3%) | 0.06 |
| **≥2, n(%)** | 340 (68.5%) | 1,795 (72.4%) | 0.08 |
| **Received any COVID-19 vaccine before index date, n(%)** | 365 (73.6%) | 1,952 (78.7%) | 0.12 |
| **Received COVID-19 vaccine within 6 weeks before index date, n(%)** | 41 (8.3%) | 283 (11.4%) | 0.11 |
| **Received ≥1 dose of any mRNA vaccine, n(%)** | 363 (73.2%) | 1,937 (78.1%) | 0.11 |
| **Received ≥1 dose of Pfizer/BioNTech Comirnaty vaccine, n(%)** | 271 (54.6%) | 1,572 (63.4%) | 0.18 |
| **Received ≥1 dose of Moderna Spikevax vaccine, n(%)** | 191 (38.5%) | 815 (39.2%) | 0.12 |
| **Received ≥1 dose of AstraZeneca Vaxzevria vaccine, n(%)** | 22 (4.4%) | 132 (5.3%) | 0.04 |
| **Recent SARS-CoV-2 PCR test before case death date** | | | |
| **Never tested positive before, n(%)** | 448 (90.3%) | 2,277 (91.8%) | 0.05 |
| **Remote prior positive test (>90 days), n(%)** | 33 (6.7%) | 184 (7.4%) | 0.03 |
| **Recent prior positive test (≤90 days), n(%)** | 15 (3.0%) | 19 (0.8%) | 0.17 |
| **Number of SARS-CoV-2 PCR tests prior to case death date** | | | |
| **Mean ± SD** | 1.32 ± 2.86 | 1.27 ± 3.32 | 0.02 |
| **Median (IQR)** | 1 (0-2) | 0 (0-1) | 0.08 |

* std = standardized difference

^1^ Cells with <6 individuals are suppressed to reduce the risk of re-identification as per ICES contractual obligations with data providers.
