# Supplementary material for: Association between COVID-19 vaccination and sudden death in apparently healthy younger individuals: A population-based case-control study
Source: PLoS Med. 2026 Mar 19;23(3):e1004924. doi: 10.1371/journal.pmed.1004924 (PMC13001984; doi:10.1371/journal.pmed.1004924)
Supplement: S6 Table — The analysis adjusted for age and quarterly rate of sudden death among unvaccinated individuals. (DOCX) [file pmed.1004924.s007.docx]

**S6 Table. The relative incidence (with 95% confidence interval) of sudden death in vaccinated individuals within the post COVID-19 vaccination risk period (6-weeks after the vaccination date for each dose received) compared to the remainder of the observation period. The analysis adjusted for age and quarterly rate of sudden death among unvaccinated individuals.**

|  | **Relative Incidence** | **95% Confidence Interval** | **P value** |
| --- | --- | --- | --- |
| **Dose 1** | 0.87 | 0.54 – 1.40 | 0.57 |
| **Dose 2** | 0.94 | 0.57 – 1.57 | 0.82 |
| **Dose 3** | 0.87 | 0.37 – 2.05 | 0.75 |
