# Supplementary figures and images for: Association between COVID-19 vaccination and sudden death in apparently healthy younger individuals: A population-based case-control study
Source: PLoS Med. 2026 Mar 19;23(3):e1004924. doi: 10.1371/journal.pmed.1004924 (PMC13001984; doi:10.1371/journal.pmed.1004924)

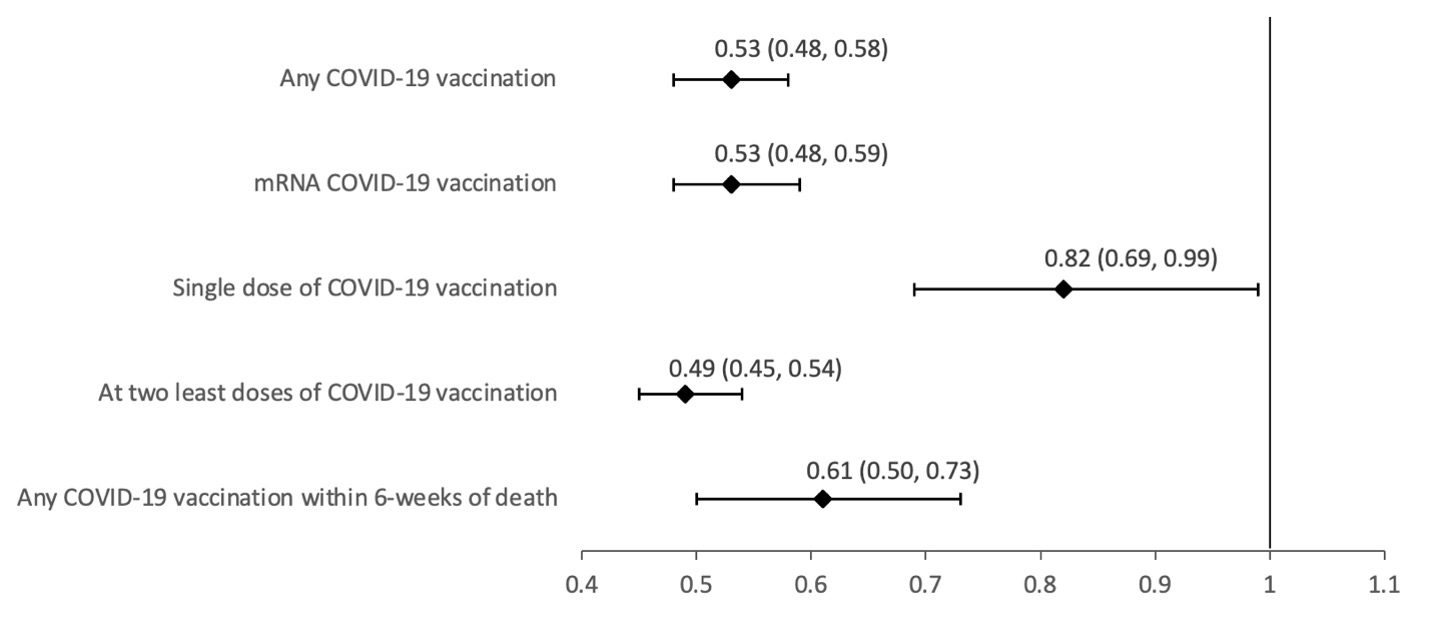

Supplement: S1 Fig — (TIFF) [file pmed.1004924.s008.tiff]

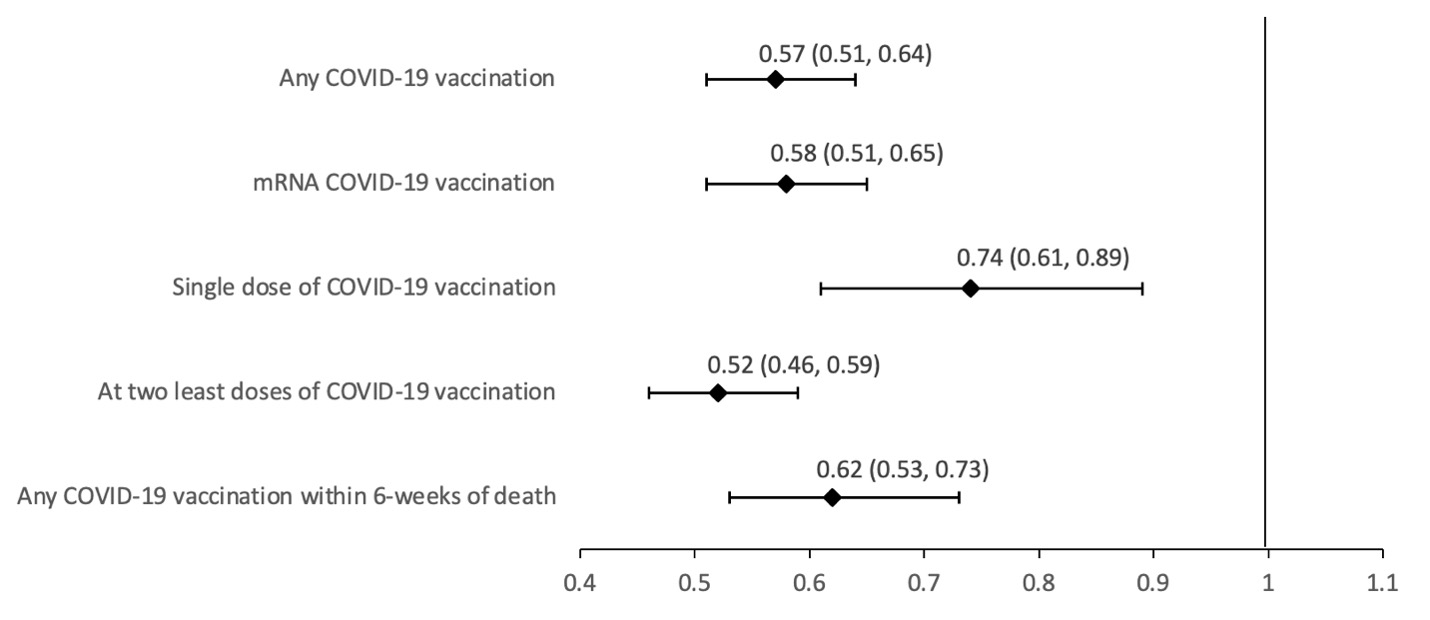

Supplement: S2 Fig — (TIFF) [file pmed.1004924.s009.tiff]

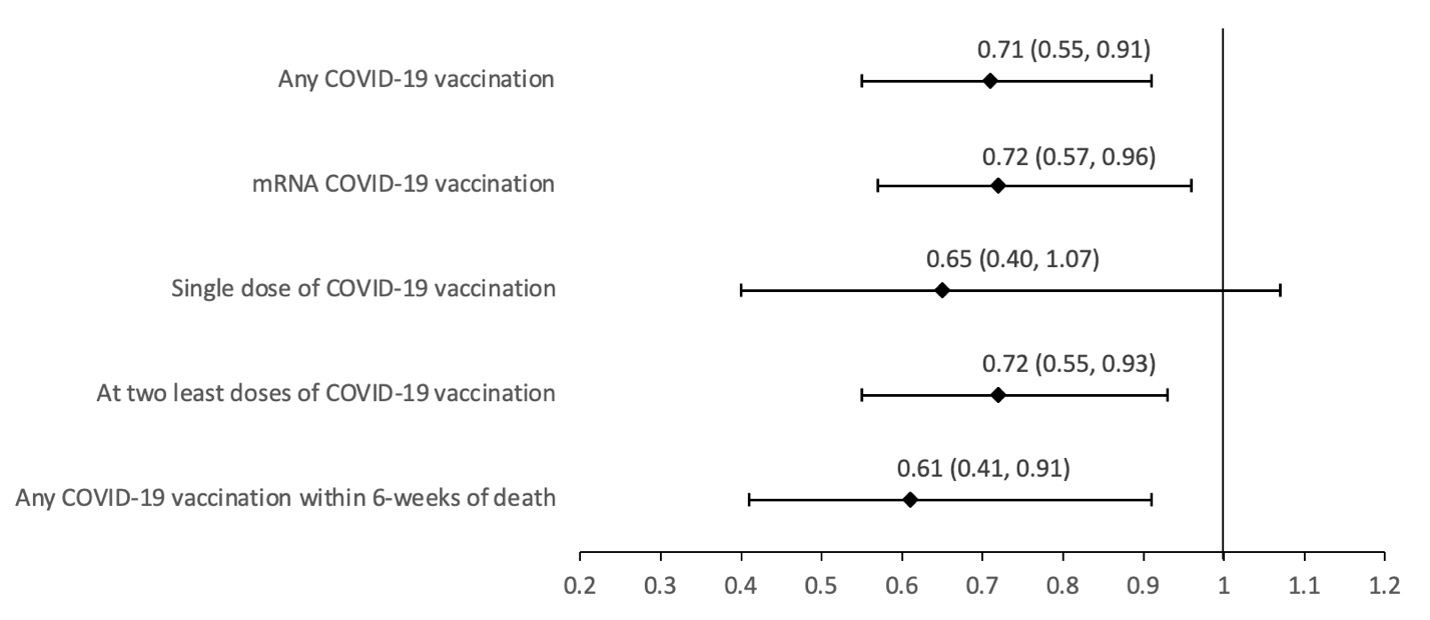

Supplement: S3 Fig — (TIFF) [file pmed.1004924.s010.tiff]
