# Supplementary material for: Association between COVID-19 vaccination and sudden death in apparently healthy younger individuals: A population-based case-control study
Source: PLoS Med. 2026 Mar 19;23(3):e1004924. doi: 10.1371/journal.pmed.1004924 (PMC13001984; doi:10.1371/journal.pmed.1004924)
Supplement: Attachment — Submitted filename: Final Response letter - Nov 2.docx [file pmed.1004924.s013.docx]

November 2, 2025

Dear Editors,

We would like to thank you and the Reviewers for your thoughtful and constructive comments on our manuscript. We have carefully revised the manuscript in response, clarifying points of methodology, expanding our discussion of limitations, and conducting an additional sensitivity analysis using a modified self-controlled case series (SCCS) design. We believe these revisions have substantially strengthened the manuscript and hope it is now acceptable for publication in PLOS Medicine. Below we provide a detailed point-by-point response to each comment.

**Editorial/Academic Editor Comments**

**Comment:** The Academic Editor thinks that you could state more clearly what is novel about your study. They agree with the comments made by reviewers #2 and #3 regarding residual confounding and encourage you to follow reviewer #2's advice and adjust the analyses.

**Response:** Thank you for this feedback. We have revised the Discussion to emphasize the novelty of our study, including its population-based design, large sample size, careful exclusion of individuals with comorbidities that predispose to sudden death, and multiple methodological approaches and sensitivity analyses. This includes our conduct of a modified SCCS analysis as suggested. We expanded the Discussion to highlight potential residual confounding, including health-seeking behavior, as an explanation for the apparent protective association. Please see Pages 18-20, lines 333-360.

**Reviewer #1**

**Comment 1:** This population-based case-control study has large sample size, reliable data source, the selection of cases is clear and well-explained, the statistical approach is straightforward, and the results are well organised, for example, it is nice to have Table 1 where the readers can have a clear understanding of the cases' demographic information as compared with the general population.

**Response:** Thank you for your positive feedback and thoughtful review of the manuscript. Please note that the prior Table 1 was moved to supplemental materials as S2 Table to address Comment 2 as well as the feedback from other reviewers.

**Comment 2:** This study has a large dataset to closely match the cases by age, sex and residence region. However, the matching by age is not clear. As by Line 134, "Controls were matched to their case according to date of birth, sex, and forward sortation area", controls should have the exact age as that of the cases. However, the variable age differs between case and control groups in the tables (Table 1, 2, S3). Please explain. If the matching is not limited to the exact date of birth, please clarify. If a case has more than 5 potential controls, what are the principles to select 5 from them? Are there cases with less than 5 matched controls?

Table 1 contains 4963 cases while Table 2 contains 4806 cases. Can the authors explain why over 150 cases were removed?

**Response:** We thank the reviewer for raising this important point which highlights the need for more clarity in our description of the data. S2 Table (previously Table 1, now moved to supplementary) lists all the people that met the case definition and the pool of potential controls who met study inclusion criteria. These 2 groups were not matched and this explains the discrepancy in age. Table 2 lists the baseline characteristics of the matched set. Of the 4,963 eligible cases, 4,806 cases were included in the analyses after each of them was matched to 5 controls (for a total of 24,030 controls). None of these 4,806 included cases had <5 controls. The remaining 157 cases were not included as they were matched to <5 controls.

This information has been clarified in the legend of the revised S2 Table and the footnote for Table 1.

S2 Table Note: The data are presented after dividing the cohort into two groups - people who met the definition for being a case, and those who were eligible for being selected as controls. Please note that the cases are not matched to controls in this table.

Table 1 Note: The data are presented after dividing the cohort into two groups: cases and matched controls.

We also revised the Results section to state (page 11, Line 228-234):

“We identified 14,664,193 Ontario residents who were alive as of April 1st, 2021. After applying exclusion criteria (**Fig 1**), we were left with 6,365,451 eligible subjects. During the accrual period, 4,963 (0.08%) subjects died and met criteria to be cases. Of these, 4,448 (89.6%) died in the prehospital setting and 515 (10.4%) died within 24 hours of presenting to a hospital or ED with a discharge diagnosis of cardiac arrest. From this pool of 4,963 cases, 4,806 (96.8%) were included in the primary case-control analysis after each was matched to 5 controls (for a total of 24,030 controls).”

**Comment 3:** The COVID pandemic triggered many abnormalities in public health and medical settings. It would be interesting to see the longitudinal trend of the sudden death rate (same definition of cases in this study) before and during COVID, and for during COVID, before and after Vaccine initiation. Having those figures - without any statistical inference - would be the icing on the cake.

**Response:** Thank you for this suggestion. While we agree this would have been interesting, it falls outside the scope of our study. We had defined the cohort based on the time period 2021-2023 and it would require a substantial undertaking to conduct the interesting analyses suggested by the reviewer.

**Comment 4:** The term "undoubtedly" in line 303 is too strong.

**Response:** This sentence has been revised as follows (page 19, Line 355-357):

“While mRNA vaccines have demonstrated an increased risk of myocarditis in young men, mortality rates following the development of postvaccine myocarditis are lower than mortality rates following COVID-19-related myocarditis or conventional myocarditis. [49].”

**Reviewer #2**

**Comment 1:** This paper addresses an important safety concern expressed largely in social media and other non-scientific fora that COVID-19 vaccines can cause sudden death in previously healthy young people. The authors use a matched case control design to test the hypothesis of an increased risk of death after COVID-19 vaccine and find no evidence in support of this.

**Response:** Thank you for your positive feedback and thoughtful review of the manuscript.

**Comment 2**: Line 187 says that prior to matching the cases were older and more likely to be male but doesn't say than whom. In table 1 the comparator column is called controls but they are just the non-cases in the eligible population. I think it is confusing to label them controls. Since this is by design a matched case control study I am unclear as to the relevance of the detail shown in Table 1. If the authors think table 1 is important to show, perhaps they can discuss its relevance otherwise it would seem more appropriate to show it in Supplementary appendix for background information.

**Response:** We agree. We have revised S2 Table (previously Table 1, now moved to supplementary) to clarify that it presents descriptive characteristics of the pool of potential cases versus the pool of potential controls. We believe it is important for the readers to understand the population from which the controls were selected. Nonetheless, we agree that the current presentation is confusing, so have moved the Table to the Supplemental Materials. This information has been clarified in the footnotes for S2 Table and the revised Table 1.

S2 Table Note: The data are presented after dividing the cohort into two groups - people who met the definition for being a case, and those who were eligible for being selected as controls. Please note that the cases are not matched to controls in this table.

Table 1 Note: The data are presented after dividing the cohort into two groups: cases and matched controls.

**Comment 2:** The numbers of cases in Table 2 which is with matched controls has dropped to 4896 from 4936 in Table 2. The authors should account for this difference.

**Response:** Addressed in the response to Reviewer 1, Comment 2.

**Comment 3:** Despite restricting the population under study by a number of factors that would predispose towards sudden death and matching controls on date of birth, sex and first 3 digits of the postcode, a lower proportion of cases than controls had received influenza vaccination. This would suggest residual confounding in some health care seeking or other behaviour/risk factors which would not necessarily be fully adjusted for by including receipt of influenza vaccine in the regression model. The consistency of the findings of an apparent protective effect of vaccination, with a lower OR after two than one dose is consistent with there being an unmeasured confounding variable associated with health care seeking behaviour. Given this, it would have been informative to conduct an analysis using the self-controlled cases only method to see whether the apparent protective effect was still there with a method that automatically controls for time invariant individual level confounding variables. Did the authors consider this method which is now widely used for vaccine safety studies to deal with the problem of unmeasured confounding? In any event this issue should be discussed by the authors and while they authors are correct that their study did not provide evidence of an increased risk of sudden death a small elevated risk may have been masked by the confounding.

**Response:** We thank the reviewer for this suggestion. We have performed a modified SCCS analysis including cases with ≥1 vaccine doses during the observation period from April 1, 2021 to June 30, 2023, with a 6-week post-vaccination risk interval. Relative incidence was estimated using conditional Poisson regression with age and quarterly background rate of sudden death as time-varying covariates. The results were consistent with our case-control findings and did not demonstrate an increased risk.

The Methods section of the manuscript has been revised to state (page 10, Line 203-214):

“In an additional post-hoc sensitivity analysis, we utilized a modified self-controlled case series (SCCS) method [38, 39]. This was restricted to vaccinated individuals meeting the case definition during the study period who had up to three doses of any COVID-19 vaccine. For this analysis the observation period remained the same (April 1, 2021 to June 30, 2023). We used a 6-week risk interval beginning on the vaccination date (day 0) for each dose received, with person-time for each interval included in the model as an offset. The control period was the remainder of the observation period after exclusion of the 6-week risk intervals if applicable. Vaccine exposures were modelled as first dose, second dose, or third dose. Relative incidence (RI) was estimated using conditional Poisson regression with a pseudo-likelihood approach and sandwich variance estimators to calculate Wald confidence intervals. Models were adjusted for age as a time-varying covariate and for quarterly rate of sudden death among unvaccinated individuals derived from our case-control analysis.”

The Results section of the manuscript has been revised to state (page 17-18, Line 318-321):

“The SCCS sensitivity analysis demonstrated that there was no significant difference in the rate of sudden death in the 6 weeks following first (RI 0.87; 95%CI [0.54,1.40]; p=0.57), second (RI 0.94; 95%CI [0.57,1.57]; p=0.82), or later doses (RI 0.87; 95%CI [0.37,2.05]; p=0.10) of a COVID-19 vaccine (**S6 Table**).”

**Comment 4:** Line 273. The authors say that "Our data align with recent publications that also report no association of COVID-19 vaccination with sudden death". They cite one study from England that used the SCCS method (ref 37) but this study found no evidence of a protective effect either. Neither did a separate study in England (Stowe J et al. Risk of cardiac arrhythmia and cardiac arrest after primary and booster COVID-19 vaccination in England: A self-controlled case series analysis. Vaccine X. 2023 Dec 1;15:100418 - not referenced by the authors). In contrast a case control study from India (ref 39) did find an apparent protective effect of COVID-19 vaccination. It would have been valuable if the authors had considered potential reasons for the apparent protective effect in the two case-control studies and why this was not observed in the two self controlled cases only studies. At the end of the discussion (line 317) the authors do seem to acknowledge the potential for confounding by saying that unvaccinated individuals may be less likely to seek health care encounters and to be more likely to have undiagnosed health conditions but don't then discuss what impact they think this this might have on their study results.

**Response:** We agree. We expanded the Discussion to highlight health-seeking behavior and unmeasured confounding as a possible explanation for the observed protective association (Discussion, p18 - 19, lines 333-360).

“Our data align with recent publications which report that COVID-19 vaccination is not associated with higher risk of sudden death. Previous studies from the United States and England utilized a modified SCCS design to demonstrate no significant increase in cardiac or all-cause mortality in the 4 weeks and 12 weeks after COVID-19 vaccination, respectively [41, 42]. A case-control study from India suggested a decreased likelihood of sudden death in young individuals who had received 2 or more doses of COVID-19 vaccines (OR=0.58; 95%CI [0.37, 0.92]) compared to those who had received only 1 dose (OR=1.00; 95%CI [0.73,1.36]) [43]. Our primary case-control analyses similarly demonstrated a dose-dependent protective effect, while the SCCS sensitivity analysis showed no protective effect associated with any dose of COVID-19 vaccination. It is possible that the apparent protective effective of increasing doses of COVID-19 vaccination in the case-control analyses may reflect confounding due to greater health-seeking behaviours among vaccinated individuals.

Collectively, these studies refute claims of an increased risk of sudden death after COVID-19 vaccinations [12, 13]. While some studies have suggested dose-dependent safety concerns due to higher risks of myocarditis after the second dose[44, 45], this does not align with our data and that from other large, epidemiologic analyses. Further supporting the absence of an increased risk of sudden death following COVID-19 vaccines, an analysis of mortality records of residents in Italy aged 1 to 40 years showed no increase in sudden cardiac death after the introduction of COVID-19 vaccines [46]. Indeed, rates of sudden death have decreased among athletes between the years 2002 – 2022, including the years when COVID-19 vaccines were introduced [47]. Furthermore, a study examining death certificates of young individuals between the years 2021 to 2022 found no evidence directly attributing COVID-19 vaccination to cardiac death [48]. While mRNA vaccines have demonstrated an increased risk of myocarditis in young men, mortality rates following the development of postvaccine myocarditis are lower than mortality rates following COVID-19-related myocarditis or conventional myocarditis­ [49]. Postmortem histopathologic analyses have raised potential causal relations between unexpected death and VITT following COVID-19 vaccination, [50-52] but this may be less relevant for mRNA vaccines which comprise most COVID-19 vaccines in use today."

**Reviewer #3**

**Comment 1:** The authors present a case control study of death and COVID-19 vaccination. The case-control study design is an appropriate choice. Potential confounders have been considered, and measured confounders have been accounted for by matching or adjustment. The paper is well written.

The protective effect of COVID-19 vaccination found could suggest that there remains unmeasured confounding, e.g. as a result of differential reporting of COVID-19 infection among cases and controls, but of course it is impossible to be certain. It is noted as a limitation in the discussion that rapid antigen tests could not be captured and so unmeasured infection could not be accounted for (this would be an issue for any study design).

I agree with the overall conclusion: There is no evidence from this study that COVID-19 vaccination is associated with an increased risk of cardiac death. Given the potential for unmeasured confounding, I think that the overall conclusions are fair and are not overstated.

**Response:** We thank the reviewer for their supportive comments. We expanded the Discussion to highlight health-seeking behavior and unmeasured confounding as a possible explanation for the observed protective association (Discussion, p18-19, lines 333-360).

**Additional Editorial Requirements**

--------------------------------------------------------- --- General editorial requests:

(Note: not all will apply to your paper, but please check each item carefully)

*	We ask every co-author listed on the manuscript to fill in a contributing author statement, making sure to declare all competing interests. If any of the co-authors have not filled in the statement, we will remind them to do so when the paper is revised. If all statements are not completed in a timely fashion this could hold up the re-review process. If new competing interests are declared later in the revision process, this may also hold up the submission. Should there be a problem getting one of your co-authors to fill in a statement we will be in contact. Please do not add or remove authors without first discussing this with the handling editor. You can see our competing interests policy here: <http://journals.plos.org/plosmedicine/s/competing-interests>.

*	Please upload any figures associated with your paper as individual TIF or EPS files with 300dpi resolution at resubmission; please read our figure guidelines for more information on our requirements: <http://journals.plos.org/plosmedicine/s/figures>. While revising your submission, please upload your figure files to the PACE digital diagnostic tool, <https://pacev2.apexcovantage.com/>. PACE helps ensure that figures meet PLOS requirements. To use PACE, you must first register as a user. Then, login and navigate to the UPLOAD tab, where you will find detailed instructions on how to use the tool. If you encounter any issues or have any questions when using PACE, please email us at [PLOSMedicine@plos.org](mailto:PLOSMedicine@plos.org).

*	Please ensure that the paper adheres to the PLOS Data Availability Policy (see <http://journals.plos.org/plosmedicine/s/data-availability>), which requires that all data underlying the study's findings be provided in a repository or as Supporting Information. For data residing with a third party, authors are required to provide instructions with contact information (web or email address) for obtaining the data. Please note that a study author cannot be the contact person for the data. PLOS journals do not allow statements supported by "data not shown" or "unpublished results." For such statements, authors must provide supporting data or cite public sources that include it.

*	We expect all researchers with submissions to PLOS in which author-generated code underpins the findings in the manuscript to make all author-generated code available without restrictions upon publication of the work. In cases where code is central to the manuscript, we may require the code to be made available as a condition of publication. Authors are responsible for ensuring that the code is reusable and well documented. Please make any custom code available, either as part of your data deposition or as a supplementary file. Please add a sentence to your data availability statement regarding any code used in the study, e.g. "The code used in the analysis is available from Github [URL] and archived in Zenodo [DOI link]" Please review our guidelines at <https://journals.plos.org/plosmedicine/s/materials-software-and-code-sharing> and ensure that your code is shared in a way that follows best practice and facilitates reproducibility and reuse. Because Github depositions can be readily changed or deleted, we encourage you to make a permanent DOI'd copy (e.g. in Zenodo) and provide the URL.

*	ETHICS STATEMENT: Please include the approval number and details on consent procedures (also, if waived).

FORMATTING - GENERAL

*	Abstract: Please structure your abstract using the PLOS Medicine headings (Background, Methods and Findings, Conclusions). Please combine the Methods and Findings sections into one section.

*	At this stage, we ask that you include a short, non-technical Author Summary of your research to make findings accessible to a wide audience that includes both scientists and non-scientists. The Author Summary should immediately follow the Abstract in your revised manuscript. This text is subject to editorial change and should be distinct from the scientific abstract. Ideally each sub-heading should contain 2-3 single sentence, concise bullet points containing the most salient points from your study. In the final bullet point of 'What Do These Findings Mean?', please include the main limitations of the study in non-technical language. Please see our author guidelines for more information: <https://journals.plos.org/plosmedicine/s/revising-your-manuscript#loc-author-summary>.

*	Please express the main results with 95% CIs as well as p values. When reporting p values please report as p<0.001 and where higher as the exact p value p=0.002, for example. Throughout, suggest reporting statistical information as follows to improve clarity for the reader "22% (95% CI [13%,28%]; p</=)". Please be sure to define all numerical values at first use.

*	Please include page numbers and line numbers in the manuscript file. Use continuous line numbers (do not restart the numbering on each page).

*	Please cite the reference numbers in square brackets. Citations should precede punctuation.

FIGURES AND TABLES

*	Please provide titles and legends for all figures and tables (including those in Supporting Information files).

*	Please define all abbreviations used in each figure/table (including those in Supporting Information files).

*	Please consider avoiding the use of red and green in order to make your figure more accessible to those with color blindness.

SUPPLEMENTARY MATERIAL

*	Please note that supplementary material will be posted as supplied by the authors. Therefore, please amend it according to the relevant comments outlined here.

*	Please cite your Supporting Information as outlined here: <https://journals.plos.org/plosmedicine/s/supporting-information>

REFERENCES

*	PLOS uses the numbered citation (citation-sequence) method and first six authors, et al.

*	Please ensure that journal name abbreviations match those found in the National Center for Biotechnology Information (NCBI) databases (<http://www.ncbi.nlm.nih.gov/nlmcatalog/journals>), and are appropriately formatted and capitalised.

*	Where website addresses are cited, please include the complete URL and specify the date of access (e.g. [accessed: 12/06/2023]).

*	Please also see <https://journals.plos.org/plosmedicine/s/submission-guidelines#loc-references> for further details on reference formatting.

STUDY TYPE-SPECIFIC REQUESTS

*	Abstract: Please include the study design, population and setting, number of participants, years during which the study took place (enrollment and follow up), length of follow up, and main outcome measures.

*	Please ensure that the study is reported according to the RECORD guideline (available from <https://www.record-statement.org>) and include the completed checklist as Supporting Information. If you believe that the STROBE guideline is more appropriate, please use it. Please add the following statement, or similar, to the Methods: "This study is reported as per the Reporting of Studies Conducted using Observational Routinely-Collected Data (RECORD) guideline (S1 Checklist)." When completing the checklist, please use section and paragraph numbers, rather than page numbers.

*	For all observational studies, in the manuscript text, please indicate: (1) the specific hypotheses you intended to test, (2) the analytical methods by which you planned to test them, (3) the analyses you actually performed, and (4) when reported analyses differ from those that were planned, transparent explanations for differences that affect the reliability of the study's results. If a reported analysis was performed based on an interesting but unanticipated pattern in the data, please be clear that the analysis was data driven.

*	Please state in the Methods section whether the study had a prospective protocol or analysis plan. If a prospective analysis plan (from your funding proposal, IRB or other ethics committee submission, study protocol, or other planning document written before analyzing the data) was used in designing the study, please include the relevant document(s) with your revised manuscript as a Supporting Information file to be published alongside your study and cite it in the Methods section. A legend for this file should be included at the end of your manuscript. If no such document exists, please make sure that the Methods section transparently describes when analyses were planned, and when/why any data-driven changes to analyses took place. Changes in the analysis, including those made in response to peer review comments, should be identified as such in the Methods section of the paper, with rationale.

__________________________________________________

In compliance with data protection regulations, you may request that we remove your personal registration details at any time. (Use the following URL: <https://www.editorialmanager.com/pmedicine/login.asp?a=r>). Please contact the publication office if you have any questions.

Journal Requirements:

1) Thank you for stating "The funding sources had no role in the conduct of the study, decision to publish or preparation of the manuscript. We have no relevant conflicts of interest to declare."

Please amend your detailed Financial Disclosure statement. This is published with the article, therefore should be completed in full sentences and contain the exact wording you wish to be published.

i). State what role the funders took in the study. If the funders had no role in your study, please state: “The funders had no role in study design, data collection and analysis, decision to publish, or preparation of the manuscript.”

ii). If any authors received a salary from any of your funders, please state which authors and which funders.

2) Please note that the contact for accessing your data cannot be an author on the manuscript. Please therefore replace the current contact information with a third party contact, such as an institutional data access committee.

3) We do not publish any copyright or trademark symbols that usually accompany proprietary names, eg (R), (C), or TM (e.g. next to drug or reagent names). Please remove all instances of trademark/copyright symbols throughout the text, including ©Canada on line number 333, ACG® on line number 111.

If you have any questions or require assistance, please don't hesitate to get in touch via return email. To help us minimize inbox volumes, we kindly ask that you not reply to this email simply to confirm you have resubmitted your revisions.
