# Supplementary material for: Association between COVID-19 vaccination and sudden death in apparently healthy younger individuals: A population-based case-control study
Source: PLoS Med. 2026 Mar 19;23(3):e1004924. doi: 10.1371/journal.pmed.1004924 (PMC13001984; doi:10.1371/journal.pmed.1004924)
Supplement: Attachment — Submitted filename: Response Letter - January, 2026.docx [file pmed.1004924.s014.docx]

8 January 2026

Suzanne De Bruijn, PhD

Associate Editor

PLOS Medicine

Dear Dr. De De Bruijn,

**Editor comments**

- “We would like you to address all remaining concerns from Reviewer 2, including modifying the abstract.”
- We would like you to reframe your narrative in the introduction, as well as in the rest of the manuscript, to not base your research on a social media narrative.”
- “We would suggest to just state that 'there is a fear that the vaccine leads to sudden death despite lack of evidence'.”
- “Please also remove the specific examples calling people out by name.”
- “Similarly, please rephrase the bullet point in the authors summary under 'what do these findings mean' to just state your conclusion, rather than that this 'refutes a narrative'.”

Response: We have addressed Reviewer 2’s remaining concerns (including the abstract) and detailed them in the section of the responses pertaining to his/ her comments.

We have also reframed the narrative away from social media framing, removed named examples, and adjusted Author Summary wording. The revised text now reads:

Abstract introduction (page 2, lines 29 - 33):

“Background: COVID-19 vaccines can cause rare but serious adverse events such as myocarditis and immune thrombotic thrombocytopenia. Despite a lack of strong evidence, concerns have been expressed that COVID-19 vaccination might lead to sudden death in younger healthy adults. We studied the association between COVID-19 vaccination and sudden death in apparently healthy people aged 12-50 years.”

Author summary (pages 3-4, lines 69 - 97 ):

“Why Was This Study Done?

•	COVID-19 vaccines were received by a large segment of the population as part of the public health response to the pandemic

•	There are emerging concerns that COVID-19 vaccines are responsible for sudden death in younger healthy individuals despite lack of evidence to support this claim

What Did the Researchers Do and Find?

•	A case-control study was conducted involving Ontario residents aged 12 to 50 years without documented comorbidities predisposing to premature death between April 1, 2021 and June 30, 2023 to examine the association between COVID-19 vaccination and sudden death

•	The primary outcome was sudden death; the exposure of interest was any COVID-19 vaccination

•	Among 6,365,451 eligible individuals, 4,806 cases who experienced sudden death were matched to 24,030 controls who were alive on the date of sudden death for each corresponding case

•	Receipt of COVID-19 vaccination was not associated with increased odds of sudden death

What do These Findings Mean?

•	These findings do not support the hypothesis that COVID-19 vaccines increase the risk of sudden cardiac death in younger healthy adults

•	A limitation of this study was the inability to confirm the cause of out-of-hospital deaths”

Manuscript introduction (page 5, lines 117 - 120):

“Recently, concerns have been raised that healthy younger people were dying suddenly because of COVID-19 vaccination, however, this notion is not supported by any reliable scientific evidence [6-9]. Because most people living in the Western hemisphere have been vaccinated, most sudden deaths are expected to occur in previously vaccinated people.”

Manuscript conclusion (page 20, lines 515 - 516):

“These data do not support the hypothesis that COVID-19 vaccines increase the risk of sudden cardiac death.”

**Reviewer 2**

Comment 1: “. I am therefore surprised that the authors have modified the conclusion of their abstract which now states that "COVID-19 vaccination was consistently associated with a lower risk of sudden death". What is the justification for adding "consistently” when the SCCS did not show a protective effect especially as there are now grounds for concluding that the apparent protective effect from the case control study likely reflects residual confounding? A more balanced conclusion would be to omit this sentence and just say "These data do not support the hypothesis that COVID-19 vaccines increase the risk of sudden cardiac death" which was the final sentence in the authors’ original abstract. This would also bring the conclusion in the abstract in line with the Author Summary (lines 77-81).”

Response: We agree with this suggestion. The revised abstract (page 2) and the concluding segment of the author summary (page 20) now state:

“These findings do not support the hypothesis that COVID-19 vaccines increase the risk of sudden cardiac death in young healthy adults.”

Minor comments

1. “I was unable to find mention of what the acronym ICES stands for in the paper. …it would be helpful if the name of ICES was given in full with a generic weblink to the main ICES website in the reference list for readers to understand exactly what this organisation is and how it works.”

Response: We have added a full form of ICES and provided a citation for the official website (page 6, lines 168 – 169):

“The datasets were linked using unique encoded identifiers and analyzed at ICES (previously known as the Institute for Clinical Evaluative Sciences [www.ices.on.ca]) [14, 15].”

2. “Line 336 - is there a verb missing here eg "found"”

Response: We have identified the error and edited accordingly (page 19, line 477)

“we consistently found that COVID-19 vaccination was not associated with increased odds…”
