# Supplementary material for: Association between COVID-19 vaccination and sudden death in apparently healthy younger individuals: A population-based case-control study
Source: PLoS Med. 2026 Mar 19;23(3):e1004924. doi: 10.1371/journal.pmed.1004924 (PMC13001984; doi:10.1371/journal.pmed.1004924)
Supplement: Attachment — Submitted filename: Response to Editorial Requests - Jan 15.docx [file pmed.1004924.s015.docx]

January 14, 2026

Suzanne De Bruijn, PhD

Associate Editor

PLOS Medicine

Dear Dr. De De Bruijn,

We are grateful for the potential acceptance of our manuscript. Please see our responses to your editorial requests.

1) Please confirm that your abstract complies with our requirements, including format (three sections: Background, Methods and Findings, and Conclusions) and providing all the information relevant to this study type

**Response:** We confirm that our abstract complies with the requirements listed – it is presented in the required format and the word count is under 500 words.

2) Please ensure that all abbreviations are defined at first use throughout the text.

**Response:** We confirm that all abbreviations are defined at first use throughout the text.

3) Please confirm that all numbers presented in the abstract are present and identical to numbers presented in the main manuscript text.

**Response:** We confirm that all abbreviations are defined at first use throughout the text.

FUNDING STATEMENT
4) Please include initials of authors who received each award.

**Response:** This has been revised as follows (page 21, line 396):

“This work was supported by funding from the Canadian Immunization Research Network (CIRN, [https://landing.cirnetwork.ca]) through a grant from the Public Health Agency of Canada and the Canadian Institutes of Health Research (CNF 151944), and funding from the Public Health Agency of Canada, through the Vaccine Surveillance Working Party and the COVID-19 Immunity Task Force (both grants to Dr. JCK).”

5) Please add URLs to the funders website, as well as grant numbers where possible.

**Response:** We have added a URL for the Canadian Immunization Research Network (primary funder).

ETHICS
6) Include IRB approval number

**Response:** The research ethics board approval number has been added to page 11, lines 221 – 225:

“The use of most data in this project is authorized under section 45 of Ontario’s Personal Health Information Protection Act (PHIPA) and does not require review by a Research Ethics Board [38]. Ethics approval was required for the analyses excluding opioid-related deaths, which was part of our secondary case definition; this was obtained from the Women’s College Hospital Research Ethics Board (REB # 2024-0002-E).”

7) Was informed consent necessary, or was this waived? Please state so in the methods section. If it was necessary, please state whether informed consent was written or oral.

**Response:** The following statement is mentioned on page 6, lines 124 - 126:

“ICES is an independent, non-profit research institute whose legal status under Ontario’s health information privacy law allows it to collect and analyze health care and demographic data, without consent, for health system evaluation and improvement.”

OTHER
8) Please mention the fact that differences in health care seeking behaviours may lead to residual confounding as an additional limitation in the abstract.

Response: The following statement has been added to the abstract (Page 2, line 59):

“Study limitations include the inability to confirm the cause of out-of-hospital deaths and residual confounding due to differences in health-seeking behaviours for the case-control analysis.”

The limitations section on page 20, lines 372 – 373 now states:

“Lastly, differences in health care seeking behaviours may have led to residual confounding; this is expected to be less applicable for the SCCS analysis.”

9) Line 104: include ‘previously’ to make clear these data are not presented in this manuscript.

**Response:** We have added previously for further clarification on page 6, lines 104 - 107:

“In a prospective evaluation of 54 participants, we previously demonstrated that 1 in 8 people with confirmed acute symptomatic myocarditis had evidence of focal myocardial inflammation by fluorodeoxyglucose positron emission tomography/magnetic resonance imaging at two months follow-up [12].”

10) Line 109: add a sentence stating the knowledge gap: e.g. ‘sudden death in apparently healthy people has not been studied yet’.

Response: The following statement has been added to page 6, lines 112 – 113:

“There are limited data on the speculated association of COVID-19 vaccination with sudden death in healthy people.”

11) Line 328: remove ‘decisively’

**Response:** The word “decisively” has been removed on page 18, lines 333:

“Collectively, these observations refute the claim that COVID-19 vaccination increases the risk of sudden death.”

12) We appreciate that you discuss the differences in covid vaccinations after matching in detail (line 250-256); however, please also mention the difference in covid vaccines in the sentence prior to this paragraph, to avoid any possible confusion. (line 242-244: “Most differences between cases and controls were nullified, but there remained a higher prevalence of hypertension and mood/anxiety disorders, and lower documented influenza vaccination, among cases.”)

**Response:** We have moved this sentence to the end of the preceding paragraph – see page 12, lines 244 – 247.

“Most differences between cases and controls were nullified after matching, but there remained a higher prevalence of hypertension and mood/anxiety disorders and lower documented COVID-19 and influenza vaccinations among cases.”

**Technical requests**

In your Data Availability Statement, please ensure you have provided all necessary access information in complete sentences. Access information should include the name of any data repositories used as well as digital object identifiers (DOIs), accession numbers or codes, or other persistent identifiers for all relevant data. If all data has been provided within your submission files, please use the statement "All relevant data is available within the manuscript and Supporting Information files." The Data Availability Statement will appear on the published version of your manuscript.

**Response**: The following statement was provided on Page 22, Line 414:

“Although data‐sharing agreements prohibit ICES from making the data set publicly available, access may be granted to those who meet prespecified criteria for confidential access, available at https://www.ices.on.ca/DAS. The underlying analytic code is available from the authors upon reasonable request, with the understanding that the computer programs may rely on coding templates or macros that are unique to ICES and are, therefore, inaccessible or may require modification.”
